# Can Particulate Air Sampling Predict Microbial Load in Operating Theatres for Arthroplasty?

**DOI:** 10.1371/journal.pone.0052809

**Published:** 2012-12-21

**Authors:** Maria Luisa Cristina, Anna Maria Spagnolo, Marina Sartini, Donatella Panatto, Roberto Gasparini, Paolo Orlando, Gianluca Ottria, Fernanda Perdelli

**Affiliations:** Department of Health Sciences, University of Genoa, Genoa, Italy; The University of Hong Kong, Hong Kong

## Abstract

Several studies have proposed that the microbiological quality of the air in operating theatres be indirectly evaluated by means of particle counting, a technique derived from industrial clean-room technology standards, using airborne particle concentration as an index of microbial contamination. However, the relationship between particle counting and microbiological sampling has rarely been evaluated and demonstrated in operating theatres. The aim of the present study was to determine whether particle counting could predict microbiological contamination of the air in an operating theatre during 95 surgical arthroplasty procedures. This investigation was carried out over a period of three months in 2010 in an orthopedic operating theatre devoted exclusively to prosthetic surgery. During each procedure, the bacterial contamination of the air was determined by means of active sampling; at the same time, airborne particulate contamination was assessed throughout the entire procedure. On considering the total number of surgical operations, the mean value of the total bacterial load in the center of the operating theatre proved to be 35 CFU/m^3^; the mean particle count was 4,194,569 no./m^3^ for particles of diameter ≥0.5 µm and 13,519 no./m^3^ for particles of diameter ≥5 µm. No significant differences emerged between the median values of the airborne microbial load recorded during the two types of procedure monitored. Particulates with a diameter of ≥0.5 µm were detected in statistically higher concentrations (p<0.001) during knee-replacement procedures. By contrast, particulates with a diameter of ≥5 µm displayed a statistically higher concentration during hip-replacement procedures (p<0.05). The results did not reveal any statistically significant correlation between microbial loads and particle counts for either of the particle diameters considered (≥0.5 µm and ≥5 µm). Consequently, microbiological monitoring remains the most suitable method of evaluating the quality of air in operating theatres.

## Introduction

All surgical procedures carry a risk of post-operative infection, which can be particularly serious in orthopedic surgery such as joint replacement [1]. The prevention of surgical site infection (SSI) after orthopedic implant surgery is a hot topic for politicians, hospital administrators and clinicians, given the enormous amount of resources these infections consume in terms of extra costs of medications, reoperations, and prolonged hospitalization [2]. Prevention involves identifying and controlling potential sources of microbial contamination.

Factors causing surgical site infections are multivarious [3], including surgical characteristics (e.g. type of procedure, duration of the operation etc), the appropriateness of staff behaviors (limited number of personnel and restricted movements) and microbial contamination of the air (especially in clean surgery) [4], [5]. In turn, the latter factor can be, at least partially, determined by factors linked to the surgical characteristics and staff behaviors.

Several studies have proposed that the microbiological quality of the air in operating theatres be indirectly evaluated by means of particle counting, a technique derived from industrial clean-room technology standards [6], using airborne particle concentration as an index of microbial contamination [6], [7]. One of the sources of airborne particulates in operating theatres may be the surgical smoke produced by particular instruments or techniques, such as the ultrasonic scalpel, electrocautery etc, during surgical procedures.

However, the relationship between particle counting and microbiological sampling has rarely been evaluated and demonstrated in operating theatres. Seal and Clark [8] compared airborne particle counting in eight size-ranges with the numbers of bacteria-carrying particles, in ultra-clean and turbulently ventilated operating theatres. They found that the number of particles in the 5–7 µm size-range correlated significantly with microbiological contamination. Landrin et al [6] compared these two methods in empty operating theatres equipped with conventional ventilation through HEPA filters. No correlation was observed between the two methods (Spearman correlation coefficient = 0.06, P = 0.6). These authors concluded that there was no reason to replace microbiological sampling with particle counting for the routine evaluation of microbiological contamination in conventionally ventilated operating theatres.

Another study that investigated whether the density of airborne particulates could predict the density of viable airborne bacteria was conducted by Stocks et al [9] during 22 arthroplasty procedures in operating rooms with a conventional ventilation system (turbulent air flow). They found a correlation between the presence of larger particles (>5 µm) and microbial contamination, which was attributed to the capability of larger particles to carry bacteria. Thus, given the inhomogeneity of the results obtained, the literature regarding the relationship between airborne particulates and airborne microbes remains controversial.

The aim of the present study was to determine whether particle counting could predict microbiological contamination of the air in an operating theatre during 95 surgical arthroplasty procedures (hip and knee). In addition, we examined the possible correlations among the microbial load, particle counting and other variables: i.e. the frequency of use of instruments that generate particulates, door-opening rates, the number of persons present in the operating theatre and the duration of the surgical procedure.

## Methods

This investigation was carried out over a period of three months in 2010 in an orthopedic operating theatre devoted exclusively to prosthetic surgery and situated within a hospital facility in the north-west of Italy. About 1,200 hip- and knee-replacement procedures (ICD9-CM: 81.51–81.54) are performed per year in this operating theatre, which has a surface area of 35.51 m^2^ and is part of a complex that provides adequate space for reception, anesthesia, surgery, recovery and observation.

**Figure 1:**
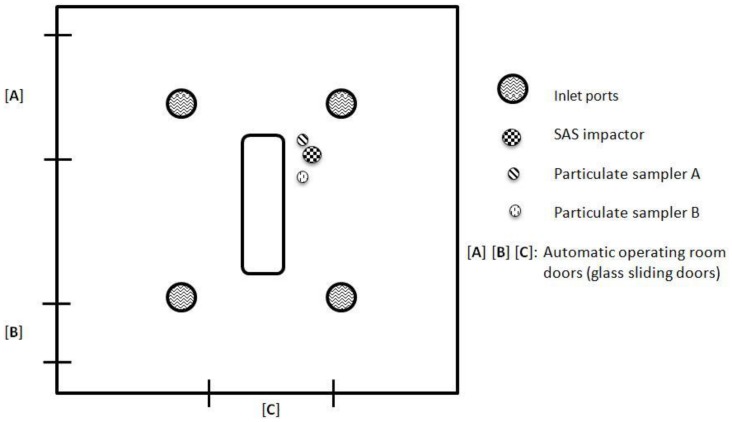
Floor plan of the operating theater, showing the positions of the samplers.

We monitored 95 surgical arthroplasty procedures: 59 hip replacements (ICD9-CM 81.51) and 36 knee replacements (ICD9-CM 81.54). During each procedure, the bacterial contamination of the air was determined by means of active sampling; at the same time, airborne particulate contamination was assessed throughout the entire procedure. Moreover, particle counts were recorded during the use of instruments able to produce surgical smoke. Figure 1 shows the floor plan of the operating theater and the positions of the air and particulate samplers.

During each operation, detailed information on the surgical procedure was collected, including the length of the procedure, the number of people present in the theatre, the door-opening rate per procedure, and the duration of use of instruments that produce surgical smoke.

At the beginning of the study, when the operating theatre was at rest, we recorded the number of air exchanges provided by the air-conditioning system and checked for any microbial contamination of the airflow from the inlet ports of the system. The operating theatre has a turbulent-flow ventilation system equipped with HEPA filters, which are 99.97% efficient in removing airborne particles of 0.3 µm or larger; the filters are replaced every 6 months and maintenance work on the system is carried out periodically. The operating room is under positive pressure in relation to the adjacent rooms (≥5 Pa); air temperature and humidity are set respectively at 17°C ±1°C and 50% ±5%.

### Determining Bacterial Contamination of the Air from the Inlet Ports and in the Centre of the Room

To determine the total airborne bacterial load, we used an SAS SUPER 100 (PBI International®) impactor equipped with RODAC plates (Ø = 55 mm) containing γ-irradiated TSA (Tryptone Soy Agar) culture medium (Biotest Italia s.r.l.). In order to sample the air in the centre of the room, the instrument was positioned in the immediate vicinity of the operating table, at a height of 1.5 m. During each procedure, a 1000 L volume of air was aspirated by means of a multi-aspiration modality; the impactor was switched on by remote control just as the skin was incised, and was switched off on completion of suturing.

To determine the bacterial load of the airflow from the inlet ports of the ventilation system in at-rest conditions, the sampler was positioned in proximity to the flow-regulators. The total volume of air sampled was 1000 L.

Plates were incubated at 37°C for 48 h before the total aerobic bacterial count was measured. Microbiological results are expressed as CFU (Colony Forming Units)/m^3^.

### Determining Contamination by Airborne Particulates

Airborne particulates (particles ≥0.5 µm and particles ≥5 µm in size) were counted by means of two light-scattering particle analyzers (Met One, Pacific Scientific Instruments, Grants Pass, OR, USA) at a flow rate of 2.8 L/min., and expressed as numbers of particles per m^3^. Both apparatuses were placed in the same position as the SAS impactor; one (Sampler A) was activated by remote control in parallel with the impactor, i.e. for the entire duration of the procedure, while the other (Sampler B), again activated by remote control, recorded the particle count both on each occasion when surgical smoke-producing instruments were used and when such instruments were not used.

### Determining the Number of Efficacious Air Exchanges

The efficacy of the air-conditioning system was assessed in at-rest conditions by measuring the decay of the concentration of tracer gas by means of a portable GA301 meter (Eco-CONTROL, Milan) connected to a computer for the collection and analysis of data, as described by Sartini et al [10].

### Statistical Analysis

Statistical analysis was carried out by means of STATA SE9™ software (Stata Corp LP - USA). The results were analyzed in terms of descriptive statistics, and the relationships between data were examined by using the non-parametric Mann-Whitney-Wilcoxon ranksum test. The rho Spearman rank correlation was used to assess the degree of association among particulate diameter ≥5 µm, particulate diameter ≥0.5 µm, bacterial load, procedure duration, percentage frequency of ultrasonic scalpel use, number of persons present and door-opening rate.

Moreover, the sensitivity and specificity of particle counting in discriminating between microbiological counting values were evaluated by means of receiver-operating characteristic (ROC) analysis.

### Ethics Statement

We didn’t need ethics approval because the study was carried out as part of routine control tests that we conduct in the operating theatres of the Hospital. As is the case of all studies conducted in the hospital environment, the General Management of the hospital approved the study protocol. The General Management is responsible for ensuring the ethical aspects of all activities of the hospital. Furthermore, the entire study was organised in accordance with a protocol agreed upon with the operating theatre teams. On entering the hospital, all patients sign an informed consent form regarding treatments in the hospital and the conditions of those treatments. Finally, the research was carried out in full respect of the Italian law on the privacy (Decreto legislativo 30 giugno 2003, n. 196).

## Results

With regard to the characteristics of the air-conditioning system, 20 efficacious air exchanges were carried out per hour. The microbial load of the airflow through the inlet ports proved to be <1 CFU/m^3^.

On considering the total number of surgical operations, the mean value of the total bacterial load in the center of the operating theatre proved to be 35 CFU/m^3^; the mean particle count measured by Sampler A was 4,194,569 no./m^3^ for particles of diameter ≥0.5 µm and 13,519 no./m^3^ for particles of diameter ≥5 µm (Table 1).

**Table 1:** Mean values, standard deviations (SD), minimum and maximum values, median and quartiles (Q1–Q3) of the total bacterial load (CFU/m^3^) and counts of particles (Sampler A) of diameter ≥0.5 µm and diameter ≥5 µm (no./m^3^) in all the procedures monitored and in each of the two types of procedure.

	Total airborne bacterial load (CFU/m^3^)		
	Hip replacement	Knee replacement	All procedures
**Mean ± SD**	35±16	34±11	35±15
**Min-max**	10–70	20–45	10–70
**Median**	35	40	40
**Q1–Q3**	25–40	22–45	25–45
	**Count of airborne particles of diameter ≥0.5 µm (no./m^3^)**		
	**Hip replacement**	**Knee replacement**	**All procedures**
**Mean ± SD**	2,538,425±1,480,054	6,908,804±3,269,100	4,194,569±3,142,263
**Min-max**	600,353–5,164,279	2,925,281–11,219,627	600,353–11,219,627
**Median**	2,418,086	6,468,327	3,127,466
**Q1–Q3**	1,294,603–3,586,316	3,760,520–9,683,440	1,630,903–5,164,279
	**Count of airborne particles of diameter ≥5 µm (no./m^3^)**		
	**Hip replacement**	**Knee replacement**	**All procedures**
**Mean ± SD**	13,915±3,592	12,868±3,488	13,519±3,571
**Min-max**	5,879–19,756	8,352–17,764	5,879–19,756
**Median**	14,520	12,207	14,520
**Q1–Q3**	12,657–16,608	9,926–16,046	11,500–16,608

Table 1 also reports the data on the microbial load and on the counts of particles of the 2 diameters (≥0.5 and ≥5 µm), subdivided by type of procedure. No significant differences emerged between the median values of the airborne microbial load (hip replacement median value: 35 CFU/m^3^; knee replacement median value: 40 CFU/m^3^) recorded during the two types of procedure monitored (p = 0.4558).

Particulates with a diameter of ≥0.5 µm were detected in statistically higher concentrations (z = −6.013; p = 0.0000) during knee-replacement procedures (median value 6,468,327 no./m^3^). By contrast, particulates with a diameter of ≥5 µm displayed a statistically higher concentration (z = 2.013, p = 0.0441) during hip-replacement procedures (median value 14,520 no./m^3^).

Table 2 reports the data on the duration of the procedures, the number of persons present in the room and door-opening rates. The difference in the median duration of the two types of procedure (hip-replacement : 40 min; knee-replacement: 45 min) proved to be statistically significant (z = −3.569; p = 0.0004).

**Table 2:** Mean, standard deviation (SD), median and range of the values of procedure length (min), number of persons present in theatre, and door-opening rate (no./min) for all surgical procedures and for hip and knee replacements.

All surgical procedures		
	Mean±SD	Median (range)
Operation length (min)	45±10	40 (35–70)
N° persons present	8±1	7 (6–10)
Door-opening rate (no./min)	0.20±0.03	0.20 (0.12–0.26)
**Hip replacement (ICD9-CM 81.51)**		
Operation length (min)	41±6	40 (35–50)
N° persons present	8±1	8 (6–10)
Door-opening rate (no./min)	0.20±0.03	0.20 (0.12–0.26)
**Knee replacement (ICD9-CM 81.54)**		
Operation length (min)	50±12	45 (40–70)
N° persons present	6±1	6 (6–7)
Door-opening rate (no./min)	0.20±0.04	0.18 (0.16–0.25)

During each operation, doors were opened a mean of 0.20 times per minute, with no statistically significant difference emerging between the two types of procedure (p = 0.2334).

All operating theatre staff wore nonwoven fabric suits; surgeons also wore highly effective isolation helmets, and instrument-handlers wore semi-integral masks, while the anesthetist and circulating nurses wore surgical masks and hair covering.

**Figure 2:**
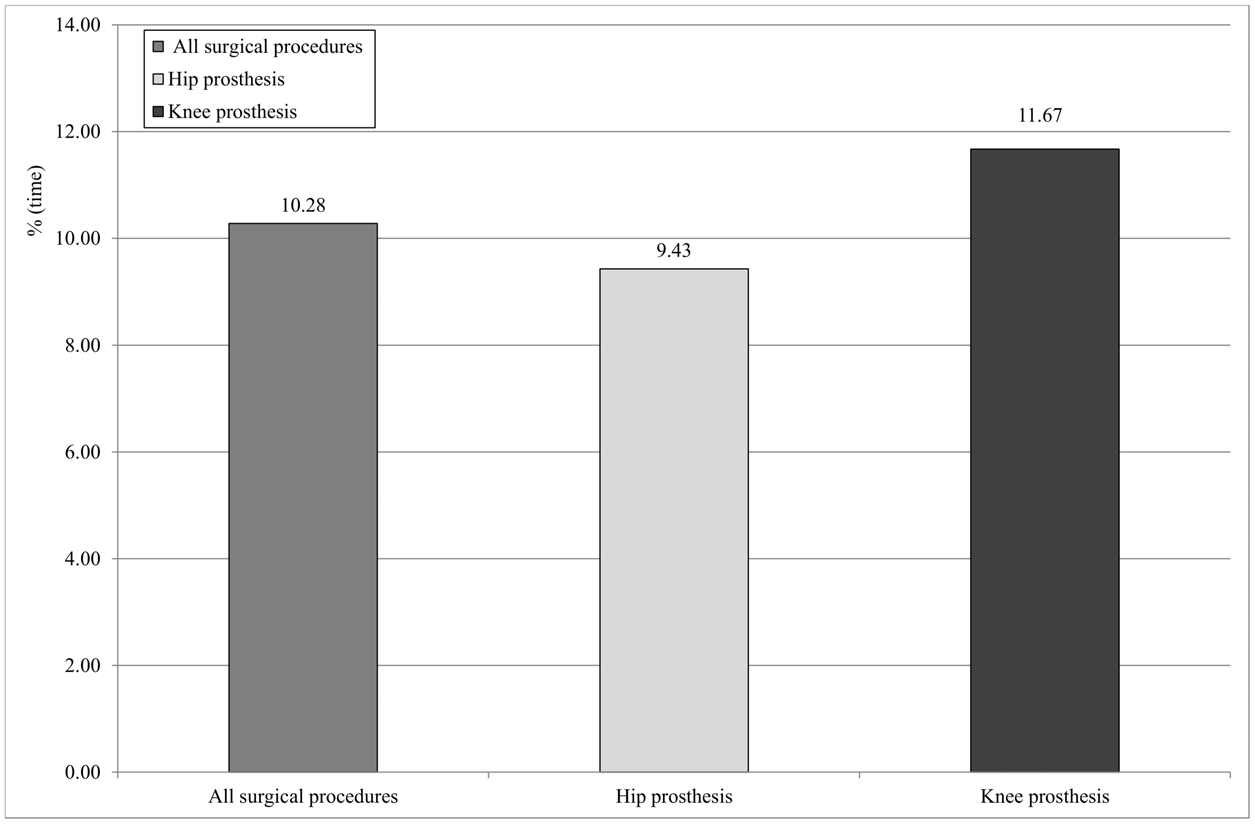
Duration of use of the ultrasonic scalpel as a percentage of operating time, subdivided by type of procedure.

Evaluation of the surgical instruments able to release surgical smoke revealed that the ultrasonic scalpel was the instrument most frequently used in both types of procedure. Figure 2 reports the duration of the use of the ultrasonic scalpel as a percentage of procedure time (calculated as minutes of use/procedure duration x 100), subdivided by type of procedure. In this regard, statistically significant differences (z = −3.145; p = 0.0017) emerged between the two types of procedure.

Airborne particulate concentration as a function of the use of the ultrasonic scalpel was evaluated by means of Sampler B. This evaluation revealed that the median concentration of particulates with a diameter of ≥0.5 µm was significantly higher (z = −4–432; p = 0.000) when this instrument was used (8,014,873 no./m^3^) than when it was not used (2,958,536 no./m^3^). A similar pattern was seen in the concentration of ≥5 µm diameter particles (13,965 no./m^3^ vs. 12,878 no./m^3^), though the difference was not statistically significant (p = 0.2019).

A highly significant correlation emerged between the percentage use of the ultrasonic scalpel and the duration of the procedures (rho = 0.8254 p<0.001). Table 3 reports of the Spearman correlation coefficients (rho) and significances (p) of the mean concentrations of particulates (≥0.5 µm and ≥5 µm) recorded by Sampler A in relation to procedure duration (min), percentage use of the ultrasonic scalpel, number of persons present and door-opening rate, both for the total number of procedures and for each procedure type (hip and knee replacement).

**Table 3:** Spearman’s correlation coefficients (Rho) and significances (p) between the mean concentration of particulate measured by Sampler A (≥5 and ≥0.5 µm) and data on procedure duration (min), ultrasonic scalpel use, number of persons present in theatre and door-opening rates, referred to all procedures and broken down by procedure type (hip and knee replacement).

	Mean concentration of particulate ≥5 µm	Mean concentration of particulate ≥0.5 µm
Procedure length (min)°°	rho = 0.1706; p = 0.0984 (*)	rho = 0.6865; **p<0.001** (*)
	rho = 0.2418; p = 0.0650 (°)	rho = 0.1888; p = 0.1521 (°)
	rho = 0.1343; p = 0.4350 (§)	rho = 0.7622; **p<0.001**(§)
% use of ultrasonic scalpel	rho = 0.1460; p = 0.1581 (*)	rho = 0.7082; **p<0.001** (*)
	rho = 0.2347; p = 0.0736 (°)	rho = 0.8612; **p<0.001** (°)
	rho = 0.5881; **p<0.001** (§)	rho = 0.7933; **p<0.001** (§)
N° persons present	rho = 0.2854; **p<0.01** (*)	rho = 0.1624; p = 0.1158 (*)
	rho = 0.4723; **p<0.001** (°)	rho = 0.2104; p = 0.1097 (°)
	rho = 0.3199; p = 0.0571 (§)	rho = 0.1464; p = 0.3943 (§)
Door-opening rate (no./min)	rho = 0.1184; p = 0.2530 (*)	rho = 0.0378; p = 0.7159 (*)
	rho = 0.2418; p = 0.0650 (°)	rho = 0.0645; p = 0.6275 (°)
	rho = 0.6895; **p<0.001** (§)	rho = 0.1389; p = 0.4192 (§)

°° skin - skin.

(*) all procedures.

(°) hip replacement.

(§) knee replacement.

The results did not reveal any statistically significant correlation between microbial loads and particle counts for either of the particle diameters considered (≥0.5 µm and ≥5 µm).

A statistically significant correlation (rho = 0.2344 p<0.05) emerged between the number of persons present in the operating theatre and the airborne microbial load during both types of procedure.

On the basis of ROC analysis, no particulate count value for the diameters of ≥0.5 and ≥5 µm can predict a microbiological count higher than 20 CFU/m^3^ (ROC area = 0.4661 and 0.1080, respectively). The choice of this cut-off was determined by the fact that the national standard reference value for operating theatres with turbulent-flow ventilation (180 CFU/m^3^) has never been reached; the most suitable cut-off value for our data proved to be the Italian and British standard value indicated for operating theatres in which ultraclean surgery is performed, despite the fact that laminar-flow ventilation is recommended.

## Discussion

During surgical procedures, dust particles, textile fibers, skin scales and respiratory aerosols loaded with viable microorganisms are released from the surgical team and patient into the surrounding air of the operating room [11]. Moreover, the use of some particular instruments during surgical procedures increases the dispersion of particulates in the air. The size of such particles closely depends on the particular instruments used. Indeed, various investigations have revealed that the electrocautery releases the smallest particles, with a mean aerodynamic size of 0.07 µm, whereas laser tissue coagulation produces larger particles (0.31 µm) and the largest particles are generated by the ultrasonic scalpel (0.35–6.5 µm) [12].

The aim of the present study was to determine whether the concentration of the airborne particulates could predict microbial contamination during surgical operations. Particle counting could offer several advantages over microbiological sampling. Indeed, microbiological sampling is time-consuming and the results may only be available after several days [6]. Another critical point concerning microbiological sampling is the absence of uniform standardized sampling procedures, which prevents data collected in different countries from being compared. In addition, a range of instruments are used in volumetric air-sampling, which hampers the correlation of results on account of their variability [3].

Conversely, particle counting is less demanding and yields immediate results. However, the information obtained is indirect [6]. This method enables results to be compared with reference values applicable to classes of maximum particle concentration. Most industrialized countries have set their own standards, based on measuring the presence of particles of varying sizes and number; many of these standards have been amended to conform to the International Standards Organization (ISO) 14644 [3], [13].

Some similar studies [6], [8], [9] have yielded results which conflict with one another, thus leaving the question unresolved.

With regard to microbial contamination in operating theatres, it is well known that the number of airborne bacteria depends on several factors, such as the number of people present in the room and their activities and behavior.

Few countries have set bacterial threshold limits in conventionally-ventilated operating theatres, although most recommend 20 air exchanges per hour, so as not to exceed values of 50–150 colony forming units (CFU)/m^3^ of air during surgical operations.

In some countries, the concentration should not exceed 180 CFU/m^3^ for an average 5-min period during activity [3].

In France [14], the microbiological limits are more restrictive than in Italy [15] and the UK, with values of ≤20 CFU/m^3^ and ≤5 CFU/m^3^ being indicated for turbulent and unidirectional airflows, respectively [16].

In the present study, the mean values of the airborne microbial load (35 CFU/m^3^) proved to be below the standard values for conventionally-ventilated [15] operating theatres in Italy. However, they exceeded the values proposed in some countries, such as France, and the generally accepted level of airborne microbial contamination during arthroplasty (10 CFU/m^3^) [10]. The values that we recorded prove to be higher than those reported in other studies of the same types of surgical procedure [9], [17].

Our analysis of the quality of the air provided by the ventilation system revealed the microbial load of <1 CFU/m^3^; it can therefore be supposed that the contamination detected was produced during surgical activity.

With regard to particle counting, the results obtained revealed mean values that exceeded the European ISO 14644 Standard limits for ISO 7 clean-rooms (352×10^3^≥0.5 µm particles/m^3^ and 2,930≥5 µm particles/m^3^ ) [13]. Moreover, the concentration values recorded in the present study, with regard to both sizes of particulates, differed from those reported by other similar studies conducted on conventionally ventilated operating theatres. However, it should be pointed out that these studies also differed considerably among themselves, again with regard to both sizes of particulates [9], [18].

It may reasonably be supposed that the utilization of surgical instruments that produce surgical smoke contributed to the high concentration of particulates. This supposition is supported by the fact that, with regard to particulates with a diameter of ≥0.5 µm, we found a significantly higher concentration (p<0.001) when the ultrasonic scalpel was being used than when it was not. Moreover, a statistically significant correlation emerged between the percentage use of this instrument and the mean concentration of ≥0.5 µm particles, and between procedure duration and the mean concentration of ≥0.5 µm particles. On the basis of these findings, it may be hypothesized that, as the duration of the procedure increases, so also does the use of the ultrasonic scalpel, i.e. the device responsible for the production of these particles.

For what concerns the heavier particles (≥5 µm), a correlation with the percentage use of the ultrasonic scalpel emerged only with regard to knee-replacement procedures. This could be explained by the fact that, as a proportion of the total particulates released by the ultrasonic scalpel, which is known to produce particulates in the 0.35–6.5 µm range, particulates with a diameter of ≥0.5 µm may prevail; by this token, the heavier particles could account for a smaller proportion of the total, becoming more detectable only after prolonged use of the device.

With regard to the correlation between ≥5 µm particles and the number of persons present in the room, the results obtained could be explained in terms of the fact that such particulates also includes the skin debris, the dimensions of which vary from 2.5 to 20 µm [18], and which are constantly being released by both theatre staff and patients. Moreover, the number of persons present influences the amount of movement taking place in the room, which, as already pointed out in other papers [18], tends to raise any dust that has already settled. Likewise, this can explain the correlation between microbial concentration and the number of persons present in the room.

In conclusion, this study showed that the use of surgical instruments that produce surgical smoke, such as the ultrasonic scalpel, can markedly contribute to the production of airborne particulates. However, neither fraction of these particulates (≥0.5 µm or ≥5 µm) displayed any correlation with the microbial load in either of the types of procedure considered.

However, one of the limitations of the present research is that only two particulate fractions were studied. We cannot therefore rule out the possibility that there may be a correlation between the number of particles/m^3^ and CFU/m^3^ if particulate fractions in narrower size-ranges are considered, as has been reported by Stocks et al. [9]. Moreover, further investigations should be carried out in operating theaters equipped with laminar-flow ventilation systems, an issue which has not yet been sufficiently addressed in studies of this kind.

Until these issues have been settled, microbiological monitoring remains the most suitable method of evaluating the quality of air in operating theatres.
